# Comparison between dopaminergic and non-dopaminergic neurons in the VTA following chronic nicotine exposure during pregnancy

**DOI:** 10.1038/s41598-018-37098-1

**Published:** 2019-01-24

**Authors:** Renee F. Keller, Tina Kazemi, Andrei Dragomir, Yasemin M. Akay, Metin Akay

**Affiliations:** University of Houston, Department of Biomedical Engineering, Houston, TX 77204 USA

## Abstract

Exposure to nicotine during pregnancy through maternal smoking or nicotine replacement therapy is associated with adverse birth outcomes as well as several cognitive and neurobehavioral deficits. Several studies have shown that nicotine produces long-lasting effects on gene expression within many brain regions, including the ventral tegmental area (VTA), which is the origin of dopaminergic neurons and the dopamine reward pathway. Using a well-established rat model for perinatal nicotine exposure, we sought to investigate altered biological pathways using mRNA and miRNA expression profiles of dopaminergic (DA) and non-dopaminergic (non-DA) neurons in this highly-valuable area. Putative miRNA-gene target interactions were assessed as well as miRNA-pathway interactions. Our results indicate that extracellular matrix (ECM) receptor interactions were significantly altered in DA and non-DA neurons due to chronic nicotine exposure during pregnancy. They also show that the PI3K/AKT signaling pathway was enriched in DA neurons with multiple significant miRNA-gene targets, but the same changes were not seen in non-DA neurons. We speculate that nicotine exposure during pregnancy could differentially affect the gene expression of DA and non-DA neurons in the VTA.

## Introduction

Maternal smoking during pregnancy is associated with adverse birth outcomes including cognitive and neurobehavioral deficits^1–3^. The results from several studies have suggested that the exposure to nicotine, the major addictive and psychoactive component of tobacco, from nicotine replacement therapy may carry risks to the offspring similar to maternal smoking during pregnancy. To investigate these underlying mechanisms, we used an established rat model of perinatal nicotine exposure using a moderate to heavy dose of nicotine^4,5^. To date, several studies have used this animal model to elucidate the genetic, biochemical, behavioral, and electrophysiological alterations that occur in response to nicotine exposure at different time points of development in different regions of the brain and by gender^3,6,7^. Due to the complexity of these contrasts, we have specifically focused on the category of neuron type: dopaminergic (DA) and non-dopaminergic (non-DA) neurons in the VTA following perinatal nicotine exposure without separating for gender differences in order to investigate more universal alterations.

Due to the addictive nature of nicotine, the brain regions of interest are part of the mesocorticolimbic pathway also known as the reward pathway. This pathway is a network of dopaminergic (DA) neurons connecting the ventral tegmental area (VTA) to the striatum, nucleus accumbens (NAc), and prefrontal cortex (PFC). In the VTA, there are three main neuron types: DA, GABA, and glutamate, which interact with each other to inhibit and activate the release of DA in other regions of the brain. Under normal behavior, DA neurons are inhibited by input from GABA neurons and activated by input from glutamate neurons. After perinatal nicotine exposure, this relationship is altered, as shown by nicotine-induced decreases in dopamine release in the NAc in adolescent rats^8^. Nicotine modulates nicotinic acetylcholine receptors (nAChRs), which are ligand-gated ion channels distributed through the central and peripheral nervous system. In the VTA, nicotine directly activates DA neurons through these receptors as well as indirectly via glutamate and GABA neurons^9–11^. Following perinatal nicotine exposure, studies have found decreased expression of nAChR subunits in the VTA as well as a decrease in the number of DA neurons^5,12,13^. These findings demonstrate specific changes that occur during development in response to chronic nicotine exposure during development. While previous studies investigating perinatal nicotine exposure have investigated biological pathways that are modulated by nicotine, none to date have explored the VTA^14–16^.

While extensive research has been conducted on epigenetic, genetic, and behavioral changes in response to perinatal nicotine exposure, less information exists on alterations that occur in the miRNome. Although there are several studies that have investigated microRNA (miRNA) changes in response to other drugs of abuse including cocaine and methamphetamine^17–19^, there are very few studies investigating miRNome expression in response to chronic nicotine exposure during pregnancy. Therefore, we previously conducted a small-scale study on specific addiction related miRNAs in DA and ND neurons in the VTA^20^. MicroRNAs (miRNAs) are short, non-coding RNA sequencing spanning about 22 nucleotides that act to regulate the expression of mRNAs by binding to the 3′ untranslated region (3′UTR) which results in translational inhibition or transcriptional repression^17,21^. They are of particular interest because a single miRNA can regulate many factors affecting gene regulatory networks, thus affecting a large downstream response^21,22^. MiRNAs have been found to modulate the effects of abusive substances and to be involved in synaptic plasticity, developing connections, and other behaviors related to addition^17,23,24^.

In the present study, we investigated RNA expression profiles of DA and non-DA neurons in the VTA following perinatal nicotine exposure due to the importance of the VTA in the mesocorticolimbic DA reward pathway as well as the changes in miRNA and mRNA expression that are modulated by nicotine. We determined significantly differentially expressed mRNAs and miRNAs as well as significant miRNA-mRNA target interactions in order to identify potential gene regulatory networks and biological pathways that result from perinatal nicotine exposure in DA and non-DA neurons in the VTA.

## Results

**Figure 1:**
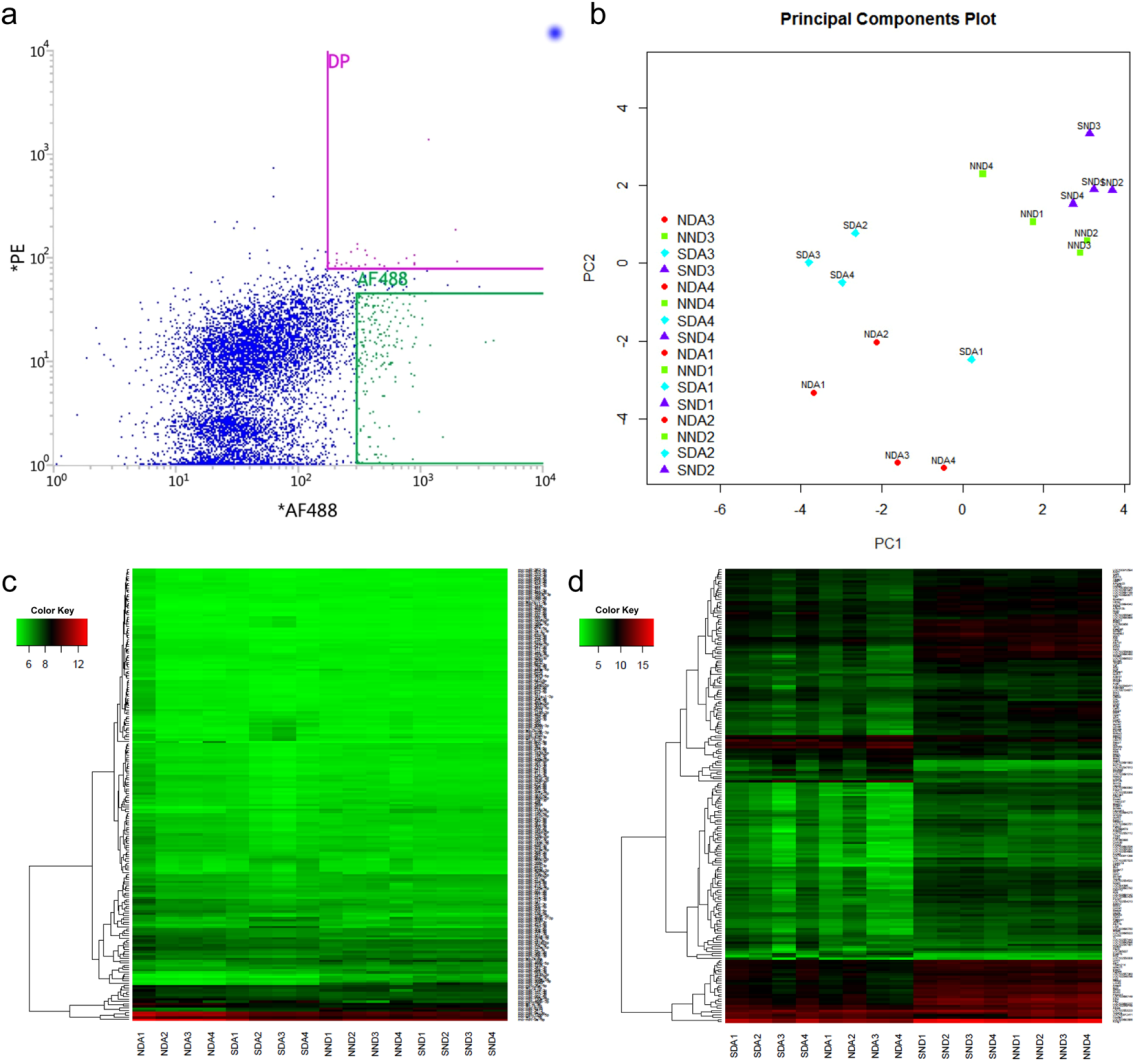
Quality assessment of DA and non-DA FACS and microarray analysis. (**a**) Typical FACS analysis results for sorting DA and non-DA neuron populations from VTA brain punches. DP shows the double-positive events indicating DA neurons by positive expression of TH and NeuN, while AF488 indicates the events positive only for the NeuN neuronal marker indicating the non-DA neuron population. The AF488 threshold was set to increase the specificity of non-DA neuron collection. (**b**) Results from principal component analysis showing clustering of the DA versus non-DA neurons as well as grouping based on saline versus nicotine treatment. Heatmaps of the microarrays showing top differentially expressed (**c**) miRNAs and (**d**) mRNAs for DA and non-DA following perinatal nicotine or saline treatment. The mRNA microarray maps also indicate a clear grouping based on neuron type.

Nicotine-treated DA and non-DA neurons were compared on the basis of transcriptome and miRNome profiles in order to find and compare the differential expression between the two neuron groups after perinatal exposure to nicotine. Rat offspring were treated with nicotine or saline (control) for up to 28 days from G6 to the second postnatal week. This period of exposure in the rat model is developmentally equivalent to the three trimesters of human pregnancy^3,25^. The VTA was then isolated from the offspring and separated into DA and non-DA neuron groups based on expression of TH (a common dopaminergic neuron marker) and NeuN (a neuronal marker) using fluorescent-activated cell sorting (FACs) (Fig. 1). Additional detail about FACs can be found in Supplemental Figure 1. The ratio of DA and non-DA neurons we collected was confirmed by reported yields from Guez-Barber *et al*.^26^ and Chung *et al*.^27^.

To investigate the differential expression profile between DA and non-DA neurons after perinatal nicotine or saline (control) exposure, we used Agilent’s SurePrint microarrays to compare gene and miRNA expression profiles. Figure 1b–d shows the results from principal component analysis (PCA) and the heatmap, which indicates a clear difference between the four sample groups tested (nicotine treated DA = NDA, saline treated DA = SDA, nicotine treated non-DA = NND, and saline treated non-DA = SND). The heatmap was generated from gene expression data showing that the top 200 significantly altered genes have different expression profiles across sample groups.

### Differential gene expression analysis of DA and non-DA neurons following perinatal nicotine treatment

Differential expression was calculated for both DA and non-DA neurons by contrasting nicotine and saline treatment. The gene expression microarray tested a total of 27,791 unique probes, of which 19,091 were annotated genes. Differentially expressed genes (DEGs) were identified based on *q*-value <0.01 (adjusted *p*-value using Benjamini-Hochberg (BH) correction) and absolute log2 fold change >1 (≥1 for upregulation, ≤−1 for downregulation). For DA neurons, the results showed 711 downregulated DEGs and 183 upregulated DEGs. For non-DA neurons, the results showed 925 downregulated DEGs and 499 upregulated DEGs.

Between the DA and non-DA neuron groups, 33 significant DEGs were regulated in the same direction. These genes may indicate similar changes that occur in all cells in the VTA in response to nicotine exposure during pregnancy. Additionally, our data found 45 significant DEGs that were oppositely regulated when comparing the DA and non-DA neuron group results (Table 1).

**Table 1:** Significant DEGs with opposite and same regulation pattern between DA and non-DA neuron results.

Gene Symbol	Entrez ID	DA	Non-DA	DA logFC	DA adj *p* val	Non-DA logFC	Non-DA adj *p* val
***Opposite Regulation***							
Pfkp	60416	−1	1	−2.065	3.858 × 10^−4^	1.127	4.981 × 10^−4^
Cstf2	683927	−1	1	−2.378	4.238 × 10^−4^	1.77	1.723 × 10^−4^
LOC102546798	102546798	−1	1	−1.667	4.657 × 10^−4^	1.464	9.518 × 10^−5^
LOC102549148	102549148	−1	1	−1.603	8.797 × 10^−3^	1.224	6.365 × 10^−4^
LOC103690149	103690149	−1	1	−1.321	1.464 × 10^−3^	1.157	7.029 × 10^−4^
LOC102547481	102547481	−1	1	−1.978	1.539 × 10^−3^	2.998	7.694 × 10^−5^
Chd2	308738	−1	1	−1.515	1.550 × 10^−3^	1.358	8.841 × 10^−4^
Abcg4	300664	−1	1	−1.386	1.587 × 10^−3^	1.266	8.357 × 10^−4^
Ndrg4	64457	−1	1	−1.322	2.772 × 10^−3^	1.549	5.995 × 10^−4^
Zmym3	317260	−1	1	−1.708	3.267 × 10^−3^	1.988	8.149 × 10^−4^
Tra2a	500116	−1	1	−1.223	3.896 × 10^−3^	1.798	3.416 × 10^−4^
Nktr	100364165	−1	1	−1.156	8.475 × 10^−3^	1.868	6.782 × 10^−4^
Nepn	309775	1	−1	1.638	8.145 × 10^−4^	−1.106	8.719 × 10^−4^
Sept11	305227	1	−1	1.020	1.464 × 10^−3^	−1.642	4.950 × 10^−5^
***Same Regulation***							
Pgap3	688174	−1	−1	−3.213	6.970 × 10^−4^	−1.266	7.735 × 10^−3^
Plxnd1	312652	−1	−1	−2.103	9.318 × 10^−4^	−1.373	1.434 × 10^−3^
Osbp2	305475	−1	−1	−1.142	2.106 × 10^−3^	−1.361	3.640 × 10^−4^
Nefh	24587	−1	−1	−1.759	3.474 × 10^−3^	−2.073	8.666 × 10^−4^
RatNP-3b	498659	−1	−1	−1.96	4.512 × 10^−3^	−3.364	2.043 × 10^−4^
LOC102553761	102553761	−1	−1	−2.092	5.354 × 10^−3^	−2.876	7.832 × 10^−4^
Ids	363513	1	1	1.005	7.766 × 10^−3^	2.132	1.628 × 10^−4^

Forty-five and 33 DEGs are shown with opposite and same regulation pattern, respectively. Regulation is noted as 1 for upregulation and −1 for downregulation. Log fold-change and BH-corrected *p*-value are listed.

### Functional enrichment analysis of DEG lists

**Figure 2:**
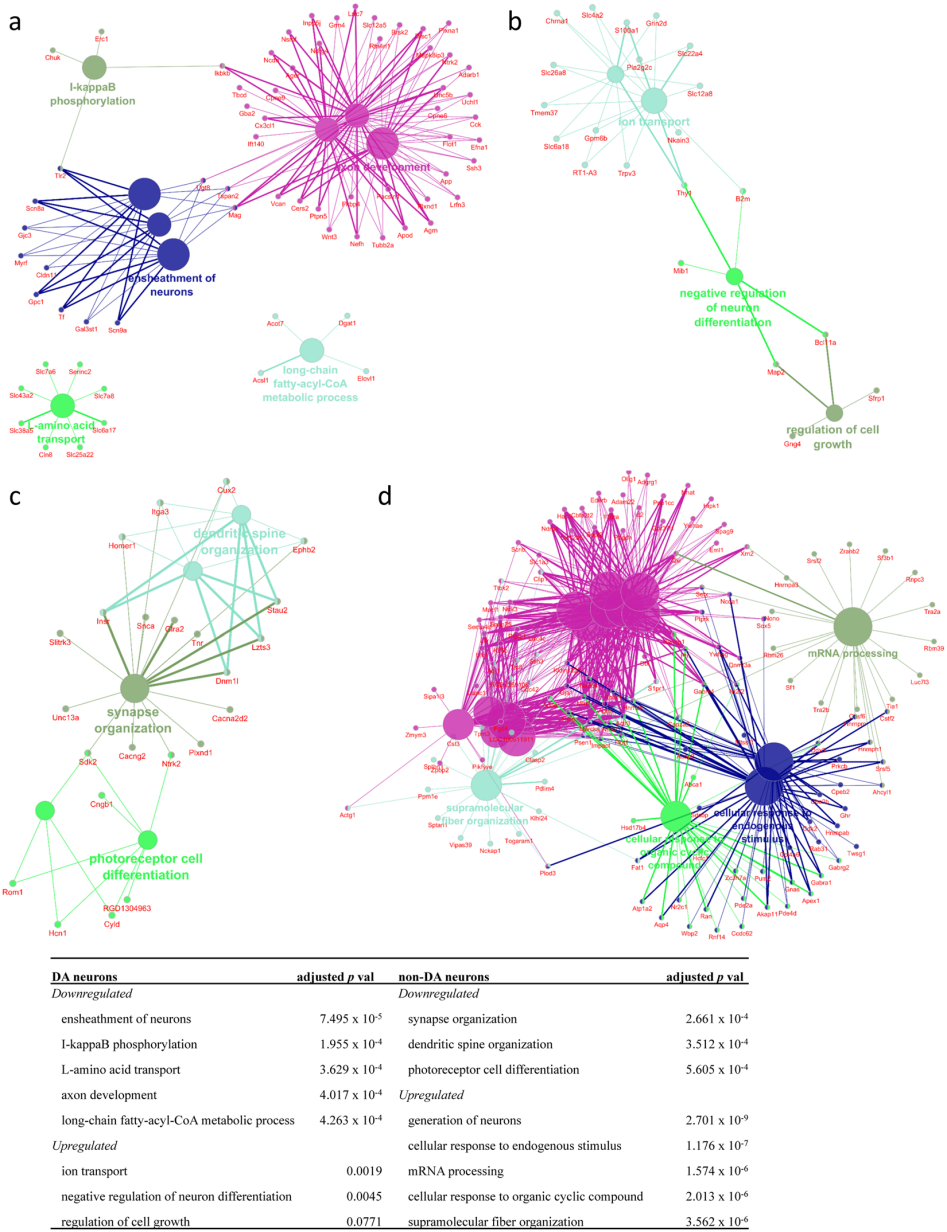
Functional enrichment analysis of DEGs from DA (**a**) down- and (**b**) up-regulated gene lists and non-DA **(c)** down- and (**d**) up-regulated gene lists. Results from GO biological processes are listed in (**e**) with BH-corrected *p*-value.

Enriched GO biological processes were investigated using the Cytoscape v3.6.0^28^ (http://cytoscape.org/) plugin ClueGO v2.5.1^29^ (Fig. 2). For the DA neuron group, the most enriched biological processes were axon development and neuron ensheathment (*p* ≪ 0.0001) for the downregulated DEGs and ion transport (*p* < 0.01) for the upregulated DEGs. For upregulated non-DA neurons, the generation of neurons was the most enriched biological process (*p* ≪ 0.0001), while for downregulated non-DA neurons, synapse organization (*p* < 0.001) was the most significantly altered biological process. In addition, we found that the expression of up-regulated DEGs in the non-DA neurons showed more dramatic changes than the changes in the DA neurons.

Next, we analyzed the DEG lists using SPIA, which compared the log fold change results from our DA and non-DA groups with signaling pathway topology in order to find significantly altered KEGG pathways^30^ (see Table 2). Two pathways were implicated in both DA and non-DA neuron results: the ECM-receptor interaction pathway was inhibited by DA DEGs and activated by non-DA DEGs; while the GABAergic synapse pathway was inhibited in both groups. Notably, the results from non-DA group showed the morphine and amphetamine addiction pathways to be inhibited.

**Table 2:** Result from SPIA analysis of the up and down-regulated DEGs for both DA and non-DA.

Name	KEGG ID	size	NDE	adj *p* val	Status
***Dopamine***					
Antifolate resistance	1523	24	3	0.003778846	Inhibited
ECM-receptor interaction	4512	61	6	0.013125414	Inhibited
GABAergic synapse	4727	77	7	0.073262087	Inhibited
Malaria	5144	40	4	0.076868383	Inhibited
PI3K-Akt signaling pathway	4151	282	15	0.086496899	Inhibited
***Non-Dopamine***					
Dilated cardiomyopathy (DCM)	5414	74	12	0.001220756	Inhibited
Morphine addiction	5032	78	12	0.010061961	Inhibited
Bile secretion	4976	55	7	0.012104698	Inhibited
Amphetamine addiction	5031	59	8	0.02023161	Inhibited
GABAergic synapse	4727	77	11	0.020302905	Inhibited
Adrenergic signaling in cardiomyocytes	4261	127	12	0.020994872	Inhibited
Leukocyte transendothelial migration	4670	95	11	0.023227661	Activated
ECM-receptor interaction	4512	61	7	0.028098198	Activated
Neurotrophin signaling pathway	4722	110	12	0.03002375	Activated
Arrhythmogenic right ventricular cardiomyopathy (ARVC)	5412	58	9	0.031670036	Inhibited
Carbohydrate digestion and absorption	4973	31	5	0.038620242	Activated
Pancreatic secretion	4972	68	7	0.041536507	Inhibited
MicroRNAs in cancer	5206	123	12	0.046085904	Inhibited
Thyroid hormone signaling pathway	4919	107	12	0.046407665	Activated
Oocyte meiosis	4114	101	12	0.049202241	Inhibited
Aldosterone synthesis and secretion	4925	68	7	0.053129335	Inhibited
cGMP-PKG signaling pathway	4022	143	13	0.062302435	Inhibited
Gastric acid secretion	4971	64	8	0.063760129	Inhibited
Salivary secretion	4970	62	7	0.068666053	Inhibited
Focal adhesion	4510	176	15	0.073336378	Activated
Regulation of actin cytoskeleton	4810	183	15	0.085677523	Inhibited
Aldosterone-regulated sodium reabsorption	4960	34	5	0.087961766	Inhibited
Hippo signaling pathway	4390	135	14	0.092760854	Inhibited

Bold pathways are common between DA and non-DA results.

### Differential miRNA expression analysis of dopamine and non-dopamine neurons following perinatal nicotine treatment

Applying a similar differential analysis scheme for miRNA expression analysis, a total of 329 annotated mature miRNAs were tested on the microarray for differential expression for each cell type. Using a *q*-value <0.05, we found 58 upregulated differentially expressed miRNAs (DEmiRs) and 16 downregulated DEmiRs for DA neurons, while our results showed 4 upregulated DEmiRs and 4 downregulated DEmiRs for non-DA neurons. Interestingly, DEmiRs were more dysregulated in the DA group compared to the non-DA group, while the opposite interpretation was observed for DEGs.

### Integrated analysis of DEmiRs and DEGs to find enriched pathways

**Figure 3:**
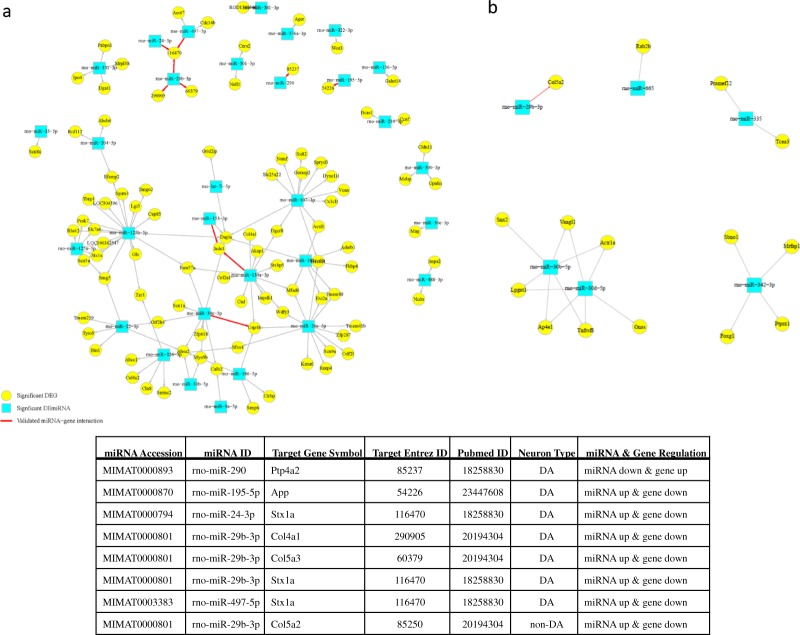
Integrated miRNA-mRNA target network. Using multiMiR, miRNA-gene target prediction was found based on significant inverse correlation of expression. For DA neuron results (**a**), 27 DEmiRs were predicted to target 89 DEGs with 125 edges. In addition, there are 7 validated miRNA-gene pairs. For ND neuron results (**b**), 6 DEmiRs were predicted to target 15 DEGs with 20 edges. The edges indicated in red are validated miRNA-gene interaction pairs based on evidence from literature. The node shapes indicate DEmiR (square) or DEG (circle). The table lists the validated miRNA-gene target pairs that are highlighted with red lines.

We processed the lists of DEmiRs and DEGs for DA and non-DA in order to find putative miRNA-gene pairs prevalent to perinatal nicotine exposure. Using multiMiR, DEmiRs and DEGs with opposite regulation were paired and compared to several databases with validated and predicted miRNA-gene target interaction scores. A total of 644 and 47 putative miRNA-gene pairs with opposite regulation were found for DA and non-DA neurons, respectively. In order to further assess target interactions and to eliminate weak correlations, pair-wise Pearson correlation was applied with BH correction. Figure 3 shows the results from our analysis, which found 125 and 42 unique miRNA-gene pairs for DA and non-DA neurons, respectively. Among the remaining significant DEmiRs, rno-miR-29b-3p and rno-miR-30b-5p were significantly enriched in both groups.

**Figure 4:**
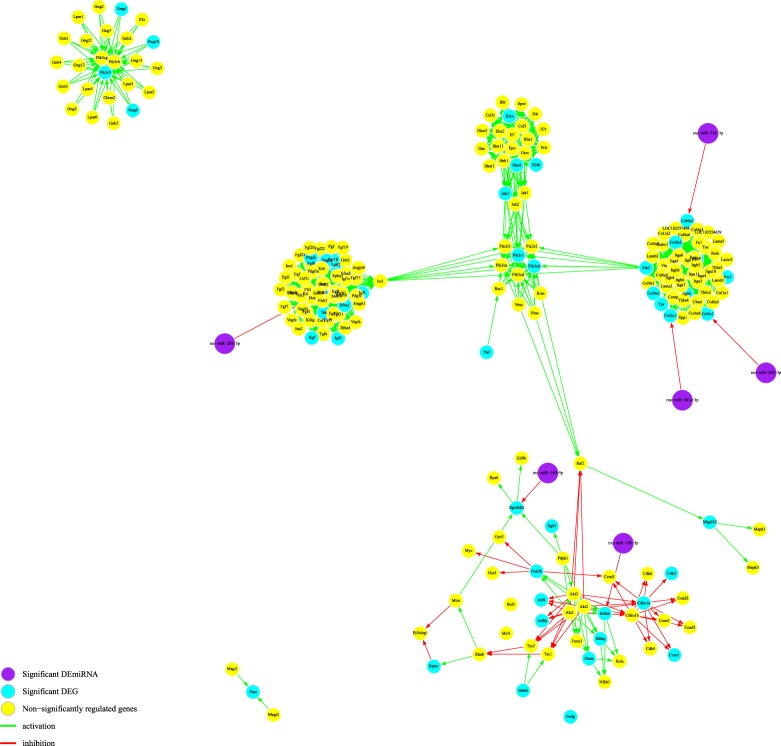
The enriched PI3K/AKT signaling pathway for DA neuron. There are five points indicating putative miRNA regulation of this signaling pathway based on the significant alteration of DEGs in the DA neuron (*p* < 0.01) and the miRNA targeting of this pathway (*p* ≪ 0.0001).

**Figure 5:**
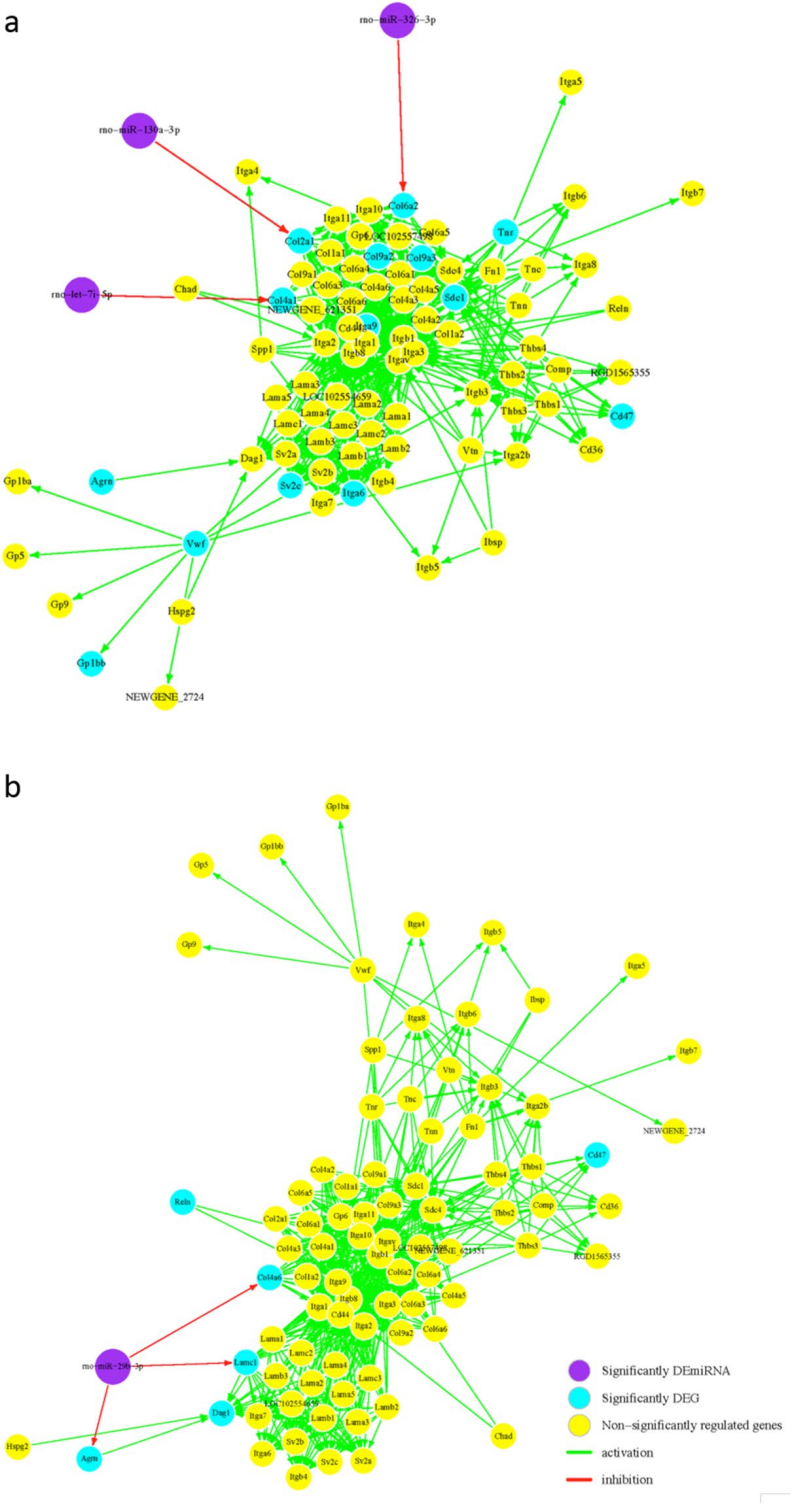
Comparing ECM-receptor interaction pathway for (**a**) DA and (**b**) non-DA neurons based on DEGs and DEmiR. (**a**) For the DA neuron, the ECM-receptor interaction pathway was altered by DEGs (*p* < 0.05) and DEmiRs (*p* ≪ 0.001) with three possible miRNA regulatory points. (**b**) For the non-DA neuron, the pathway was similarly significant for DEGs (*p* < 0.05) and DEmiRs (*p* ≪ 0.001) with one miRNA targeting many genes. Purple nodes are significant DEmiRs, blue nodes are significant DEGs, and yellow nodes are non-significantly regulated genes.

Using miRPath v.3, enriched pathways were found from the results of DEmiR-DEG pairs using the input DEmiRs and DEGs lists. Table 3 shows the pathway enrichment results from DEmiR-DEG network for DA and non-DA neurons. Due to the significance of the ECM-receptor interaction pathway in both the DA and non-DA groups, we visualized the network with putative miRNA interaction (Fig. 4). MiRNA interaction with the ECM-receptor interaction pathway was *p* ≪ 0.0001 for ND DEmiRs. For the DA neuron, the PI3K/AKT signaling pathway was significantly enriched by DEGs (*p* < 0.1) with significant miRNA interaction (*p* ≪ 0.0001) (Fig. 5).

**Table 3:** Results from miRPath v.3 for DA and non-DA neurons using DEGs and DEmiRs.

KEGG pathway	*p*-value	# genes	# miRNAs
*Dopamine*			
ECM-receptor interaction	2.264E-17	11	3
Mucin type O-Glycan biosynthesis	2.690E-17	12	14
MicroRNAs in cancer	3.990E-09	55	24
Proteoglycans in cancer	3.655E-04	57	25
PI3K-Akt signaling pathway	1.080E-03	36	5
Gap junction	0.00201	25	16
Protein processing in endoplasmic reticulum	0.00237	45	23
Renal cell carcinoma	0.00318	22	16
MAPK signaling pathway	0.00318	64	24
Neurotrophin signaling pathway	0.00386	37	19
Glioma	0.00671	19	16
Hepatitis B	0.00712	36	22
Adrenergic signaling in cardiomyocytes	0.00764	39	23
TGF-beta signaling pathway	0.01088	28	19
Cocaine addiction	0.01114	13	12
Bile secretion	0.01368	21	13
Maturity onset diabetes of the young	0.01444	9	10
N-Glycan biosynthesis	0.01444	13	16
Estrogen signaling pathway	0.01444	26	16
Ubiquitin mediated proteolysis	0.01444	37	21
Signaling pathways regulating pluripotency of stem cells	0.01444	36	21
T cell receptor signaling pathway	0.01444	30	21
Rap1 signaling pathway	0.01444	51	23
Pathways in cancer	0.01444	85	24
Chronic myeloid leukemia	0.01476	24	20
Hippo signaling pathway	0.01627	37	19
cGMP-PKG signaling pathway	0.02101	43	21
Ras signaling pathway	0.02348	50	24
Proximal tubule bicarbonate reclamation	0.02447	9	9
Osteoclast differentiation	0.03247	34	20
Choline metabolism in cancer	0.03248	28	17
Fatty acid degradation	0.04065	6	8
Wnt signaling pathway	0.04093	38	21
cAMP signaling pathway	0.04203	48	24
Aldosterone-regulated sodium reabsorption	0.04434	14	11
*Non-Dopamine*			
ECM-receptor interaction	7.510E-15	10	3
Glioma	4.110E-07	13	5
Mucin type O-Glycan biosynthesis	5.670E-06	4	2
Gap junction	8.620E-05	10	5
Glutamatergic synapse	8.620E-05	14	6
Focal adhesion	0.00519	25	5
Rap1 signaling pathway	0.00519	24	6
Arrhythmogenic right ventricular cardiomyopathy (ARVC)	0.00637	9	4
Long-term depression	0.00959	5	5
cGMP-PKG signaling pathway	0.01050	19	6
Cocaine addiction	0.01168	5	5
Morphine addiction	0.01472	7	4
Protein digestion and absorption	0.02115	13	4
Proteoglycans in cancer	0.03389	16	4

Bold pathways are common between DA and non-DA results.

### Validation of microarray data by RT-qPCR

**Figure 6:**
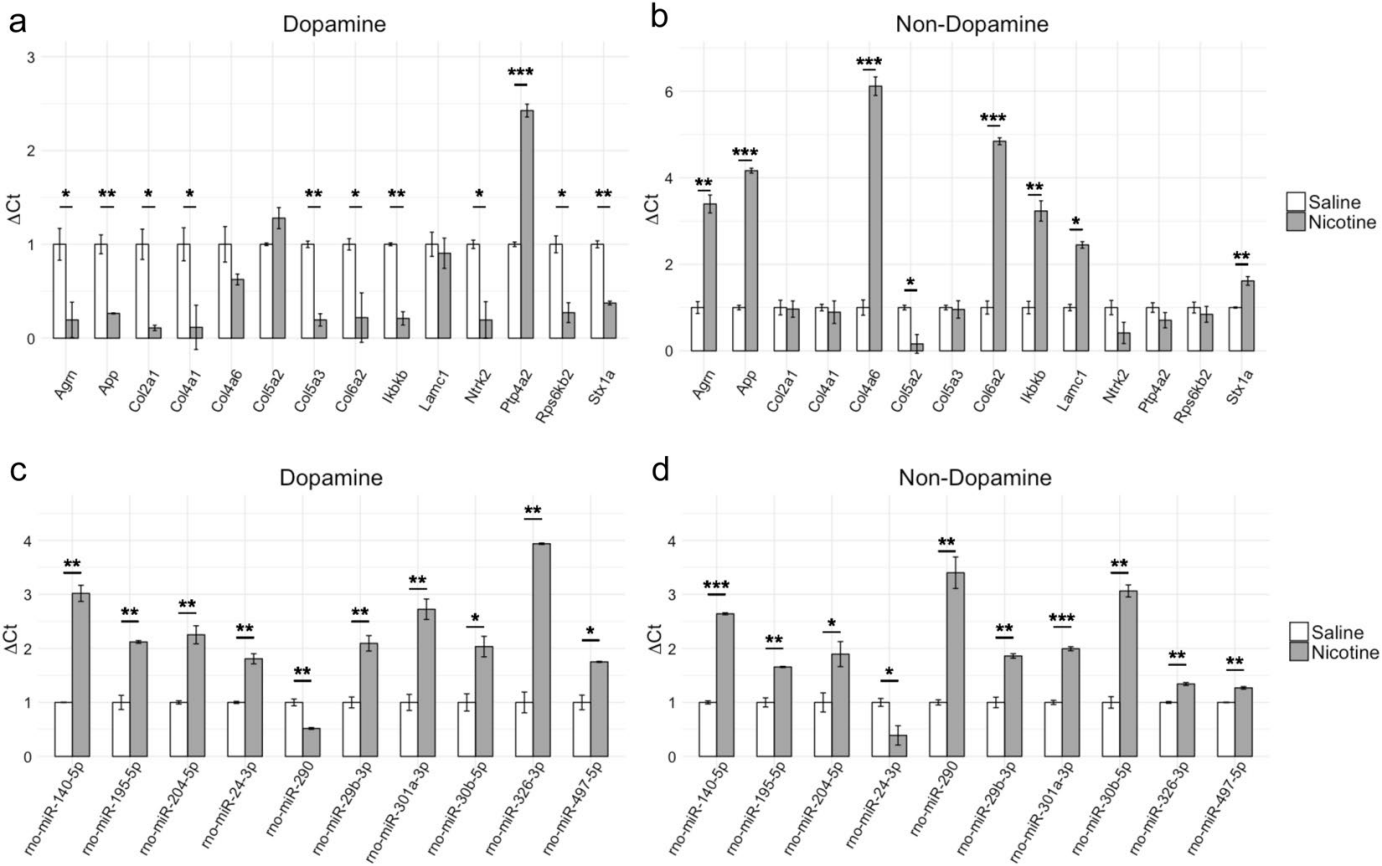
Validation of (**a,b**) mRNA and (**c,d**) miRNA microarray results by RT-qPCR. Fourteen DEGs and 10 DEmiRs were tested for DA and non-DA to assess the validity of microarray experiments. The results are shown as ΔΔCt values relative to saline-counterpart and reference primer, GAPDH for mRNA and U6 snRNA for miRNA. Significance was evaluated using Student’s t-test (n = 3) and corrected for multiple comparisons using Benjamini-Hochberg procedure with false discovery rate of 0.05 (*p < 0.05, **p < 0.01, ***p < 0.001).

The microarray results were validated for both miRNA and mRNA using the same approach detailed in Keller *et al*.^31^. Genes and miRNAs were selected based on validated miRNA-gene target interaction identified using multiMiR as well as biological interest from functional enrichment analysis. For validation of the microarrays, we chose 14 mRNAs and 10 miRNAs. All mRNAs and miRNAs tested by RT-qPCR showed the same direction of regulation, which confirmed our microarray results. The results from RT-qPCR validation are shown in Fig. 6. Reference samples used were saline-control and reference was GAPDH for mRNA testing and U6 snRNA for miRNA testing. Significance was evaluated using Student’s t-test (n = 3) and corrected for multiple comparisons using BH procedure with false discovery rate of 0.05.

## Discussion

In this study, we analyzed the differential expression of mRNA and miRNA from VTA neurons in order to compare the function enrichment analyses of DA and non-DA neurons from the VTA with respect to perinatal nicotine treatment. To investigate the effect of chronic nicotine exposure during pregnancy, we used a well-established animal model for perinatal nicotine exposure that mimics maternal smoking or nicotine replacement therapy during a full term human pregnancy^25^. We employed FACS in order to sort VTA neurons based on the expression of TH, a DA neuron marker, and NeuN, a neuronal marker, and confirmed our results against previous studies^26,27^. In summary, we found 711 downregulated and 183 upregulated DEGs for DA neurons and 925 downregulated and 499 upregulated DEGs for non-DA neurons based on *q*-value < 0.01 (BH-corrected) and absolute log2 fold change >1. For miRNA differential expression, we found 16 downregulated and 58 upregulated DEmiRs for DA neurons and 4 upregulated and 4 downregulated DEmiRs for non-DA neurons based on *q*-value <0.05 (BH-corrected). Enriched GO biological processes were analyzed to expose altered pathways in response to perinatal nicotine exposure and to reveal how nicotine alters RNA expression differently based on neuron type. Lastly, we visualized putative miRNA-gene target interactions that may highlight gene regulatory networks that are significantly modulated by exposure to nicotine during development.

Our functional enrichment analysis of DA and non-DA DEGs revealed multiple GO biological processes related to cell death and survival pathways to be significantly enriched, results that are consistent with a study by Wei *et al*., who investigated the apoptotic effects of perinatal nicotine using a pathway-focused microarray. In that study, which investigated only a panel of 29 genes using RT-qPCR, they concluded that, in adolescents, perinatal nicotine modulates gene expression in cell death and survival-related pathways, could be classified into three subgroups: growth factor, death receptor, and kinase cascade, and these alterations differed by brain region^14^. However, Wei *et al*. have not investigated the differences between DA and other types of neurons.

In the DA neuron, neuron ensheathment including myelination were significantly enriched by downregulated DEGs. A study by Cao *et al*. found that perinatal nicotine modulates myelin gene expression and shows altered expression patterns in different brain regions during different time points. Myelination is associated with addiction and psychiatric disorders that are implicated in maternal smoking during pregnancy^32^. Additionally, Takahashi *et al*. found that experimentally induced demyelination increased dopamine signaling^33^. Therefore, significant downregulation of genes involved in neuron ensheathment may result in dysregulated behavior along the DA reward pathway. The upregulated DEGs in the DA neuron were most significantly enriched in the ion transport process, which was a significantly enriched process when analyzing a list of nicotine addiction-related genes compiled by Liu *et al*.^34,35^. This implicates that the genetic alterations found in nicotine addiction studies relating to ion transport may be more significant in DA neurons of the VTA.

It was found that synapse organization was significantly altered by downregulated non-DA DEGs, which is highly important in the arrangement and microenvironment of neurons during neurodevelopment. The third trimester of human gestation and the first twelve postnatal days for rats are characterized by rapid brain growth and is a time period when nAChRs are highly involved in the formation of cortical and limbic circuitry that is essential for synaptic formation and neuronal signaling^25^. Furthermore, upregulated non-DA DEGs were highly implicated in many processes related to the generation of neurons, including morphogenesis, neurogenesis, development, and differentiation. Neuronal proliferation and death are intrinsic to normal neurodevelopment; therefore any alteration of the balance between proliferation and death may have severe results^14^. Perinatal nicotine exposure has been shown to decrease the total number of neurons in the brain and certain sub-regions including the VTA^7,12^.

Results from our miRNA analysis suggest that miRNAs are more involved in dysregulation of DA neurons compared to non-DA neurons in the VTA. The common miRNA, rno-miR-30b-5p, was implicated in several pathways, including mucin type O-glycan biosynthesis, gap junction, cGMP-PKG signaling pathway, and glutamatergic synapse pathways.

For the DA neuron, the PI3K/AKT signaling pathway was significantly enriched by DEGs with five putative DEmiR-DEG interactions. This pathway is involved in regulating cell proliferation and growth, and inhibiting cell death. Nicotine is known to trigger specific immune-related signaling pathways including PI3K/AKT signaling, which is manifested in the neuroprotective behavior attributed to nicotine^14,16,36^. Cui *et al*. found that immune pathways including PI3K/AKT signaling were modulated via Chrna7, the alpha-7 subunit of nAChR and exhibited neuroprotection against Alzheimer’s disease in animal models^16^. The putative miRNA-gene targets may shed light on possible mechanisms to induce neuroprotection against degenerative disorders like Alzheimer’s and Parkinson’s diseases. Furthermore, other abusive substances such as cocaine have been shown to modulate the PI3K/AKT signaling pathway, showing similar characteristics between perinatal nicotine exposure and drug addiction^37^.

In the pathway analysis, the ECM-receptor interaction pathway was implicated in both DA and non-DA groups, which highlights the importance of the pathway with respect to nicotine exposure and how the variation of different genes can cause similar patterns of alteration. ECM receptors are important during neurodevelopment—playing a key role in axon guidance, dendrite projection, synapse formation, and connection establishment between cells^38^. Many of these processes were significantly enriched in our analysis of GO biological processes.

In summary, we investigated significantly altered biological pathways determined by DEGs and DEmiRs. Analyzing the relationships between miRNAs and genes revealed putative miRNA-gene target interactions that may be important in regulation of biological pathways in DA and non-DA neurons in the VTA. Our results suggest that ECM-receptor interactions are significantly altered in DA and non-DA neurons due to chronic nicotine exposure during pregnancy. Additionally, the PI3K/AKT signaling pathway was enriched in DA neurons with multiple significant miRNA-gene targets, but the same changes were not seen in non-DA neurons. Further investigation of the miRNA-gene target interaction pairs needs to be conducted. The development of an interactive model to evaluate the role of these miRNAs from our predictive model in regulating gene networks and biological pathways is also necessary.

## Materials and Methods

### Animal treatment

All experiments were performed in accordance with the protocols approved by the Institutional Animal Care and Use Committee (IACUC) and the University of Houston Animal Care Operations (ACO). Pregnant female Sprague–Dawley (SD) rats (Charles River, Wilmington, MA, USA) were maintained on a 12-h light/12-h dark schedule in 22 ± 2 °C and 65% humidity. Access to standard food and water was ad libitum. Rats were acclimated to the animal facility for 72 hours before they received implanting a subcutaneous osmotic minipump (Alzet, Cupertino, CA) containing either nicotine hydrogen tartrate salt (Sigma-Aldrich, St. Louis, MO, USA) released at a rate of 6 mg/kg/day (moderate to heavy smoker), or an equal volume of saline for the control. Over a period spanning from gestational day 6 (G6) to delivery (around G21–22), the offspring received nicotine via the placenta, which readily allows nicotine to cross the placental barrier^4,39^. After birth, the offspring continue to receive nicotine via the milk which has a milk-to-plasma ratio of 2.9 ratio^4^.

Ten-to-fourteen day-old pups (male and female) were anesthetized with isoflurane before decapitation. On a VT1200 semiautomatic vibrating blade microtome (Leica, Nussloch, Eisfeld, Germany), 1 mm horizontal slices containing VTA were sliced and 1 mm biopsy punch (Integra Miltex, VWR, Radnor, PA, USA) was used to collect the VTA bilaterally. The brain punches were placed on ice in Hibernate A (Gibco, Thermo Fisher Scientific, USA) to maintain tissue viability. Brain punches from eight to ten pups from one litter were pooled into one sample with an equal number of male and female pups. We collected and analyzed four samples for each treatment group (saline and nicotine) for RNA extraction and microarray processing.

### Cell Sorting and RNA extraction

Brain punches were dissociated and sorted by FACS as reported by Guez-Barber *et al*.^26^. Briefly, brain punches were dissociated in Accutase (Gibco, Thermo Fisher Scientific, USA). Cellular debris was removed by serial filtration through decreasing cell strainers and then density centrifugation using Percoll (GE Healthcare, VWR, USA). The single cell suspension was fixed for immunolabeling by resuspension in equal parts Hibernate A and 100% ethanol, gently vortexed, and kept on ice for 15 minutes. To identify neurons, we labeled with the antibody NeuN, a neuronal marker, conjugated with Alexa Fluor 488 (NeuN/AF488, ab190195, Abcam, Cambridge, MA, USA). To identify DA neurons, we used the antibody for tyrosine hydroxylase, a dopamine specific enzyme, conjugated with phycoerythrin (Th/PE, ab209921, Abcam, Cambridge, MA, USA). Using conjugated antibodies simplifies the staining procedure by eliminating the need for a secondary antibody, which can bind unspecifically and reduces the time it takes to stain cells.

Cells were sorted on an Influx (BD Biosciences, San Jose, CA, USA) instrument at the Flow Cytometry and Cellular Imaging Core Facility (MD Anderson – South Campus, Houston, TX, USA). NeuN +/TH- populations were labeled non-DA neurons and double stained NeuN+/TH+ populations were labeled DA neurons. Total RNA was isolated using miRNeasy Micro Kit (Qiagen, Hilden, Germany) following manufacturer’s instructions, including DNAse treatment. RNA quality and quantity was established according to the optical density (OD) of each sample at 260 nm and 280 nm determined using a NanoDrop 2000 spectrophotometer (Thermo Fisher Scientific, Waltham, MA, USA). Only total RNA samples with A260/280 ratio of 1.9 or greater were used in subsequent experiments.

### Microarray Preparation, Labeling, and Hybridization

All gene and miRNA microarray reagents and kits were purchased from Agilent (Santa Clara, CA, USA) unless otherwise stated. The gene expression was profiled using a SurePrint G3 Rat Gene Expression v2 8 × 60 K microarray (ID: 074036) with 30,584 unique genes using a starting quantity of 25 ng total RNA. Samples were prepared using the One-Color Low Input Quick Amp Labeling kit with RNA Spike-Ins according to manufacturer’s instructions. Then, amplified cRNA was fragmented and prepared for hybridization using the Gene Expression Hybridization kit according to manufacturer’s instructions. Then, the slides were hybridized for 17 hours at 65 °C.

For miRNA expression, an 8 × 15 K Rat miRNA Microarray, Release 21.0 (ID: 070154) was used containing 758 mature miRNAs. For labeling and hybridization, miRNA Complete Labeling and Hyb kit with RNA Spike-Ins was used according to manufacturer’s instructions using starting quantity of 100 ng total RNA containing miRNAs. Samples were purified on a Micro Bio-Spin P-6 gel column (Bio-Rad), dried, and hybridized at 55 °C for 20 hours.

After hybridization, the slides were washed using Gene Expression Wash Buffers, according to manufacturer’s protocol. The gene and miRNA expression slides were scanned using G4900DA SureScan Microarray Scanner using appropriate protocols. Raw microarray data was extracted from microarray images using Feature Extraction (FE) Software v12.0.1.

### Data Analysis

Data analysis was performed as described previously in Keller *et al*.^31^. All pre-processing, normalization, and statistical analyses were performed in R version 3.4.2^40^ using the following packages: arrayQualityMetrics^41^ to assess quality of raw microarray data, limma^42^ to analyze mRNA microarrays, and AgiMicroRna^43^ to analyze miRNA microarrays. For the mRNA microarray experiment, feature and background outliers were removed from the data based on Agilent flags. Then, the data was background corrected using “normexp” method and quantile normalized. For the miRNA microarray experiment, raw data was preprocessed using the robust multi-array average (RMA) algorithm without background correction^44^.

For mRNA and miRNA analysis, differential expression was found using 2 × 2 factorial design comparing treatment (nicotine vs. saline) and cell type (DA vs. non-DA). A linear model was fitted with a factor containing the four conditions (nicotine treated DA = NDA, saline treated DA = SDA, nicotine treated non-DA = NND, and saline treated non-DA = SND). Differential expression between two conditions was calculated using a moderated t-test. Correction for multiple testing for all microarray analyses was performed using Benjamini-Hochberg correction using false discovery rate of 0.05.

### Functional enrichment analysis of DEGs

Functional enrichment analysis was performed on differentially expressed genes using DAVID v6.8^45,46^ and the ClueGO v2.5.1^29^ plugin for Cytoscape v3.6^28^. Gene ontology (GO) term and Kyoto Encylopedia of Genes and Genomes (KEGG) pathways were both included in the analysis. ClueGO performed single cluster analysis to create network of functionally-related terms biological processes using the list of significantly differentially expressed genes. To find significance, the p-value was calculated by performing a two-sided hypergeometric test and corrected for multiple testing using Benjamini-Hochberg method.

In order to identify enriched pathways, we used SPIA, which identifies the most relevant KEGG pathways by comparing a genelist of DEGs and their log fold change with signaling pathway topology^30^. Results from this analysis were corrected for multiple testing using the BH method.

### miRNA-mRNA integrated analysis

As previously described in Keller *et al*.^31^, multiMiR^47,48^ was used to identify predicted and validated miRNA-gene target pairs based on inversely correlated regulation of DEmiRs and DEGs. multiMiR is a comprehensive compilation of predicted and validated miRNA-gene target interactions from 14 external databases. For each predicted miRNA-gene pair, we performed pair-wise Pearson correlation analysis on expression levels of the gene and miRNA in order to assess probability of the significance. We removed pairs that did not meet the following parameters: r < −0.5 and *q* < 0.05, according to Mach *et al*.^49^. From these results we compiled a miRNA-gene network containing all DEmiRs and DEGs with significant correlation. The miRPath v.3^50^ tool was used to perform functional enrichment analysis on the miRNA-gene network with BH correction for multiple comparisons.

### Quantitative RT-qPCR validation of microarray data

All reagents and kits for quantitative reverse transcription polymerase chain reaction (RT-qPCR) were purchased from Applied Biosystems (Thermo Fisher Scientific, Carlsbad, CA, USA) unless otherwise stated. As previously described in Keller *et al*.^31^, 14 genes and 10 miRNAs were chosen for validating microarray data using RT-qPCR. Total RNA was isolated from FACS samples for each experimental group as described above. For gene expression validation, cDNA was prepared using High Capacity cDNA Reverse Transcription Kit according to manufacturer’s instructions. For miRNA expression validation, cDNA was prepared and preamplified using TaqMan Advanced miRNA cDNA Synthesis Kit according to manufacturer’s instructions. For gene validation and miRNA validation, quantitative PCR (qPCR) was carried out using TaqMan Fast Advanced Master Mix and corresponding TaqMan Assay on a StepOnePlus Real-Time PCR System using the following parameters: 2 min at 50 °C, 2 min at 95 °C, 40 cycles of 1 sec at 95 °C and 20 sec at 60 °C. For each sample, there were three biological samples (n = 3) and each reaction was prepared in triplicate. Comparative Ct method was used to find the relative quantity of the target genes or miRNAs compared to saline counterpart. GAPDH and U6snRNA were used as the reference gene and miRNA, respectively. Significance was computed using Student’s t-test with BH-correction for multiple testing with false discovery rate of 0.05.

## Supplementary information

Supplementary Information

## References

[1] Agrawal A (2010). The effects of maternal smoking during pregnancy on offspring outcomes. Prev. Med. (Baltim)..

[2] Oliff HS, Gallardo KA (1999). The effect of nicotine on developing brain catecholamine systems. Front. Biosci..

[3] Abbott LC, Winzer-Serhan UH (2012). Smoking during pregnancy: lessons learned from epidemiological studies and experimental studies using animal models. Crit. Rev. Toxicol..

[4] Matta SG (2007). Guidelines on nicotine dose selection for *in vivo* research. Psychopharmacology (Berl)..

[5] Gold AB, Keller AB, Perry DC (2009). Prenatal exposure of rats to nicotine causes persistent alterations of nicotinic cholinergic receptors. Brain Res..

[6] Ross EJ, Graham DL, Money KM, Stanwood GD (2015). Developmental Consequences of Fetal Exposure to Drugs: What We Know and What We Still Must Learn. Neuropsychopharmacology.

[7] Schneider, T. In *Negative Affective States and Cognitive Impairments in Nicotine Dependence* 91–110, 10.1016/B978-0-12-802574-1.00006-5 (Elsevier, 2017).

[8] Kane VB, Fu Y, Matta SG, Sharp BM (2003). Gestational Nicotine Exposure Attenuates Nicotine-Stimulated Dopamine Release in the Nucleus Accumbens Shell of Adolescent Lewis Rats. J. Pharmacol. Exp. Ther..

[9] Lüscher C, Malenka RC (2011). Drug-Evoked Synaptic Plasticity in Addiction: From Molecular Changes to Circuit Remodeling. Neuron.

[10] Feduccia AA, Chatterjee S, Bartlett SE (2012). Neuronal nicotinic acetylcholine receptors: neuroplastic changes underlying alcohol and nicotine addictions. Front. Mol. Neurosci..

[11] Pierce RC, Kumaresan V (2006). The mesolimbic dopamine system: The final common pathway for the reinforcing effect of drugs of abuse?. Neurosci. Biobehav. Rev..

[12] Chen H, Parker SL, Matta SG, Sharp BM (2005). Gestational nicotine exposure reduces nicotinic cholinergic receptor (nAChR) expression in dopaminergic brain regions of adolescent rats: Gestational nicotine reduces adolescent nAChR expression. Eur. J. Neurosci..

[13] Doura, M. B., Gold, A. B., Keller, A. B. & Perry, D. C. Adult and periadolescent rats differ in expression of nicotinic cholinergic receptor subtypes and in the response of these subtypes to chronic nicotine exposure. *Brain Res* **1215** (2008). 10.1016/j.brainres.2008.03.056 PMC2493527 18474362

[14] Wei J (2011). Gestational nicotine treatment modulates cell death/survival-related pathways in the brains of adolescent female rats. Int. J. Neuropsychopharmacol..

[15] Cao J (2011). Modulation of cell adhesion systems by prenatal nicotine exposure in limbic brain regions of adolescent female rats. Int. J. Neuropsychopharmacol..

[16] Cui W-Y, Li MD (2010). Nicotinic Modulation of Innate Immune Pathways Via α7 Nicotinic AcetylcholineReceptor. J. Neuroimmune Pharmacol..

[17] Bosch PJ, Benton MC, Macartney-Coxson D, Kivell BM (2015). mRNA and microRNA analysis reveals modulation of biochemical pathways related to addiction in the ventral tegmental area of methamphetamine self-administering rats. BMC Neurosci..

[18] Quinn RK (2015). Distinct miRNA expression in dorsal striatal subregions is associated with risk for addiction in rats. Transl. Psychiatry.

[19] Quinn, R. K. *et al*. Temporally specific miRNA expression patterns in the dorsal and ventral striatum of addiction-prone rats. *Addict. Biol*. 1–12, 10.1111/adb.12520 (2017). 10.1111/adb.12520 28612502

[20] Keller RF (2017). Investigating the Effect of Perinatal Nicotine Exposure on Dopaminergic Neurons in the VTA Using miRNA Expression Profiles. IEEE Trans. Nanobioscience.

[21] Siegel G, Saba R, Schratt G (2011). microRNAs in neurons: manifold regulatory roles at the synapse. Curr. Opin. Genet. Dev..

[22] Yang, Z. & Li, M. D. In *eLS* **2**, 1–15 (John Wiley & Sons, Ltd, 2017).

[23] Chandrasekar V, Dreyer J-L (2011). Regulation of MiR124, Let-7d, and MiR-181a in the accumbens affects the expression, extinction, and reinstatement of cocaine-induced conditioned place preference. Neuropsychopharmacology.

[24] Hogan EM (2014). miRNAome analysis of the mammalian neuronal nicotinic acetylcholine receptor gene family. RNA.

[25] Dwyer JB, McQuown SC, Leslie FM (2009). The dynamic effects of nicotine on the developing brain. Pharmacol. Ther..

[26] Guez-Barber D (2012). FACS Purification of immunolabeled cell types from adulat rat brain. J. Neurosci. Methods.

[27] Chung S (2014). Impact of Circadian Nuclear Receptor REV-ERBα on Midbrain Dopamine Production and Mood Regulation. Cell.

[28] Shannon, P. *et al*. Cytoscape: a software environment for integrated models of biomolecular interaction networks. *Genome Res*. 2498–2504, 10.1101/gr.1239303.metabolite (2003). 10.1101/gr.1239303 PMC403769 14597658

[29] Bindea, G. *et al*. ClueGO: A Cytoscape plug-in to decipher functionally grouped gene ontology and pathway annotation networks. *Bioinformatics*, 10.1093/bioinformatics/btp101 (2009). 10.1093/bioinformatics/btp101 PMC2666812 19237447

[30] Tarca AL (2009). A novel signaling pathway impact analysis. Bioinformatics.

[31] Keller RF, Dragomir A, Yantao F, Akay YM, Akay M (2018). Investigating the genetic profile of dopaminergic neurons in the VTA in response to perinatal nicotine exposure using mRNA-miRNA analyses. Sci. Rep..

[32] Cao J, Dwyer JB, Gautier NM, Leslie FM, Li MD (2013). Central myelin gene expression during postnatal development in rats exposed to nicotine gestationally. Neurosci. Lett..

[33] Takahashi N, Sakurai T, Davis KL, Buxbaum JD (2011). Linking oligodendrocyte and myelin dysfunction to neurocircuitry abnormalities in schizophrenia. Prog. Neurobiol..

[34] Sun J, Zhao Z (2010). Functional Features, Biological Pathways, and Protein Interaction Networks of Addiction-Related Genes. Chem. Biodivers..

[35] Liu X (2015). Prioritizing Genes Related to Nicotine Addiction Via a Multi-source-Based Approach. Mol. Neurobiol..

[36] Kanlikilicer P, Dragomir A, Zhang D, Akay YM, Akay M (2017). The Age-Related Effect Of Nicotine On The Expression Of Neuroprotective Genes In Ventral Tegmental Area And Substantia Nigra. IEEE Life Sci. Lett..

[37] Doura MB, Luu TV, Lee NH, Perry DC (2010). Persistent gene expression changes in ventral tegmental area of adolescent but not adult rats in response to chronic nicotine. Neuroscience.

[38] Kerrisk, M. E., Cingolani, L. A. & Koleske, A. J. In 101–131, 10.1016/B978-0-444-63486-3.00005-0 (2014).

[39] Wickström R (2007). Effects of nicotine during pregnancy: human and experimental evidence. Curr. Neuropharmacol..

[40] Team, R. C. R: A language and environment for statistical computing. (2017).

[41] Kauffmann A, Gentleman R, Huber W (2009). arrayQualityMetrics—a bioconductor package for quality assessment of microarray data. Bioinforma. Appl. NOTE.

[42] Ritchie, M. E. *et al*. limma powers differential expression analyses for RNA-sequencing and microarray studies. *Nucleic Acids Res*. **43** (2015). 10.1093/nar/gkv007 PMC4402510 25605792

[43] Lopez-romero, P. AgiMicroRna: Processing and Differential Expression Analysis of Agilent microRNA chips. *R package version 2.28.0* (2017). 10.1186/1471-2164-12-64 PMC3037903 21269452

[44] López-Romero P (2011). Pre-processing and differential expression analysis of Agilent microRNA arrays using the AgiMicroRna Bioconductor library. BMC Genomics.

[45] Huang DW, Sherman BT, Lempicki RA (2009). Systematic and integrative analysis of large gene lists using DAVID bioinformatics resources. Nat. Protoc..

[46] Wei Huang D, Sherman BT, Lempicki RA (2009). Bioinformatics enrichment tools: paths toward the comprehensive functional analysis of large gene lists. Nucleic Acids Res..

[47] Ru Y (2014). The multiMiR R package and database: integration of microRNA–target interactions along with their disease and drug associations. Nucleic Acids Res..

[48] Ru, Y., Mulvahill, M., Mahaffey, S. & Kechris, K. multiMiR: Integration of multiple microRNA-target databases with their disease and drug associations. *R package version 0.98.0.2* (2017).

[49] Mach N (2016). Integrated mRNA and miRNA expression profiling in blood reveals candidate biomarkers associated with endurance exercise in the horse. Sci. Rep..

[50] Vlachos IS (2015). DIANA-miRPathv3.0: deciphering microRNA function with experimental support. Nucleic Acids Res..

