# Supplementary material for: Comparison between dopaminergic and non-dopaminergic neurons in the VTA following chronic nicotine exposure during pregnancy
Source: Sci Rep. 2019 Jan 24;9:445. doi: 10.1038/s41598-018-37098-1 (PMC6345743; doi:10.1038/s41598-018-37098-1)
Supplement: Supplementary file 1 — Supplementary Information [file 41598_2018_37098_MOESM1_ESM.pdf]

Comparison between dopaminergic and non-dopaminergic neurons in the VTA following chronic nicotine exposure during pregnancy

Renee F. Keller[1], Tina Kazemi[1], Andrei Dragomir[1], Yasemin M. Akay[1], and Metin Akay[1,*]

[1]University of Houston, Department of Biomedical Engineering, Houston, TX, 77204, USA
[*]Corresponding author: makay@uh.edu

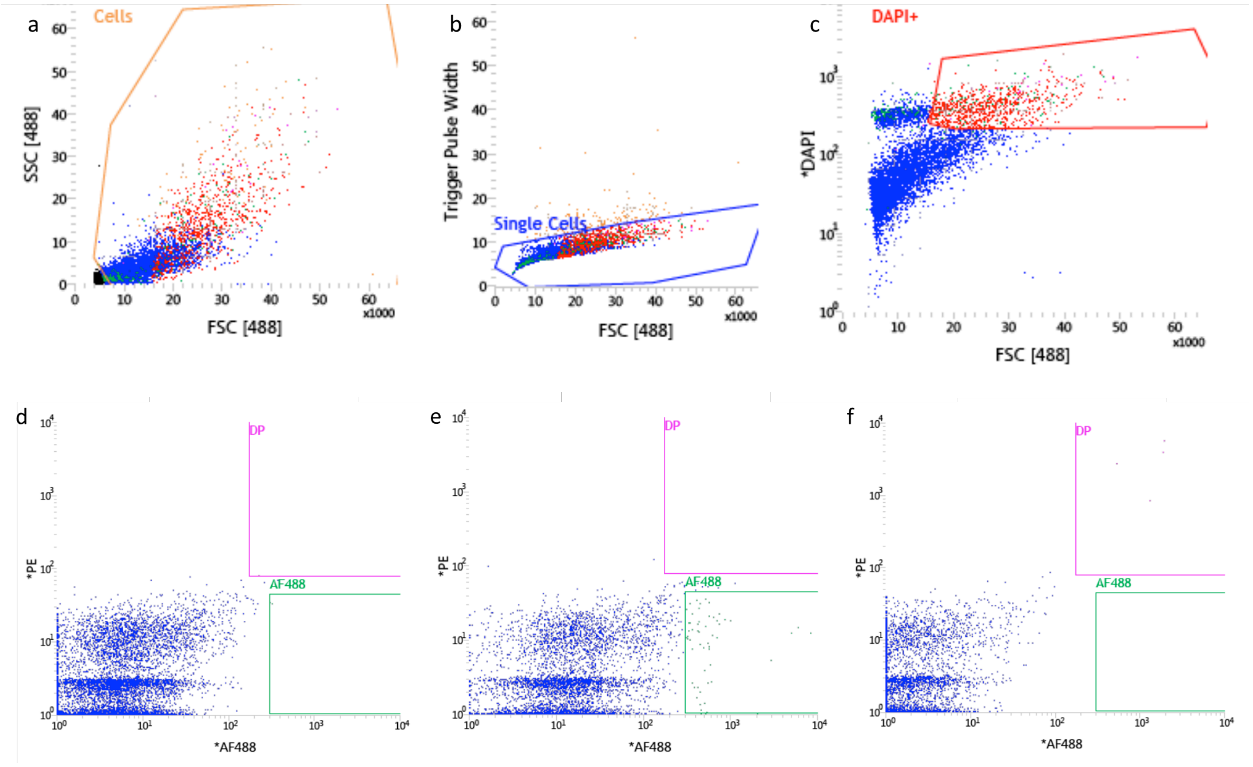

Supplemental Figure 1. Flow cytometry results from a representative sample used for determining the sorting criteria. (a-b) A typical light scatter plot showing side scatter (SSC) vs. forward scatter (FSC) of our samples. Forward scatter represents the size of the event, while side scatter represents granularity. (c) To gate for single cell events that were viable, we used DAPI staining which indicates intact cells. Furthermore, thresholds were established using the following reference samples that were (d) unstained, (e) only AF488, and (f) only PE.
